# Increased Functional Connectivity Between Medulla and Inferior Parietal Cortex in Medication-Free Major Depressive Disorder

**DOI:** 10.3389/fnins.2018.00926

**Published:** 2018-12-10

**Authors:** Lizhu Luo, Kunhua Wu, Yi Lu, Shan Gao, Xiangchao Kong, Fengmei Lu, Fengchun Wu, Huawang Wu, Jiaojian Wang

**Affiliations:** ^1^The Clinical Hospital of Chengdu Brain Science Institute, MOE Key Laboratory for Neuroinformation, University of Electronic Science and Technology of China, Chengdu, China; ^2^Department of MRI, The First People’s Hospital of Yunnan Province, The Affiliated Hospital of Kunming University of Science and Technology, Kunming, China; ^3^The Department of Medical Imaging, The First Affiliated Hospital of Kunming Medical University, Kunming, China; ^4^School of Foreign Languages, University of Electronic Science and Technology of China, Chengdu, China; ^5^The Affiliated Brain Hospital of Guangzhou Medical University (Guangzhou Huiai Hospital), Guangzhou, China; ^6^Guangdong Engineering Technology Research Center for Translational Medicine of Mental Disorders, Guangzhou, China

**Keywords:** major depressive disorder, resting-state functional connectivity, brainstem, subregions, medulla, inferior parietal cortex

## Abstract

Emerging evidence has documented the abnormalities of primary brain functions in major depressive disorder (MDD). The brainstem has shown to play an important role in regulating basic functions of the human brain, but little is known about its role in MDD, especially the roles of its subregions. To uncover this, the present study adopted resting-state functional magnetic resonance imaging with fine-grained brainstem atlas in 23 medication-free MDD patients and 34 matched healthy controls (HC). The analysis revealed significantly increased functional connectivity of the medulla, one of the brainstem subregions, with the inferior parietal cortex (IPC) in MDD patients. A positive correlation was further identified between the increased medulla-IPC functional connectivity and Hamilton anxiety scores. Functional characterization of the medulla and IPC using a meta-analysis revealed that both regions primarily participated in action execution and inhibition. Our findings suggest that increased medulla-IPC functional connectivity may be related to over-activity or abnormal control of negative emotions in MDD, which provides a new insight for the neurobiology of MDD.

## Introduction

Major depressive disorder (MDD) is mainly characterized with sustained negative affect and diminished positive affect. Neuroimaging studies, especially those using resting-state functional magnetic resonance imaging (rs-fMRI), have revealed that MDD is a disease with aberrant interactions of brain networks (Buchanan et al., 2014; Mulders et al., 2015; Smith, 2015; Wu et al., 2016; Kang et al., 2017; Sun et al., 2018; Wang C. et al., 2018). Using resting-state functional connectivity (RSFC) analyses, abnormalities in cortical networks including the default mode network (DMN), central executive network (CEN), and salience network (SN) have been well delineated (Mulders et al., 2015; Wang J. et al., 2018). Moreover, recent studies have demonstrated that the functional connectivities in frontostriatal and limbic circuits could be effective indicators to subdivide MDD into four biotypes, which in turn also serve as good predictors of treatment response in MDD (Drysdale et al., 2017; Wager and Woo, 2017). Although the high-order functional network abnormalities have been well studied, basic functions related brain areas including the brainstem in MDD remain largely unknown.

The brainstem, together with limbic and cortical areas, compose a vertical-integrative and interconnected hierarchical system that is critical in emotion as well as kinds of cognition (Geva and Feldman, 2008; Abe et al., 2010; Lee et al., 2015; Nishijo et al., 2018). Brainstem lesions could have a crucial impact on higher-level functions of cortical regions, such as attention, executive function and self-regulation (Geva and Feldman, 2008; Nishijo et al., 2018). The brainstem is called “the emotional brainstem” due to its critical role in human emotions by integrating its subregions into three major networks involving in emotional sensory, motor and modulatory (Venkatraman et al., 2017). Moreover, the brainstem is a primary source of neurotransmitter innervations such as serotonergic and dopaminergic ones that are critically associated with a wide range of brain functions, and their dysregulations of fronto-limbic circuits and the hypothalamic-pituitary-adrenal (HPA) axis in MDD have been reported (Aihara et al., 2007; Song et al., 2014; Han et al., 2017). These findings point to a key role of the brainstem in the pathophysiology of MDD.

However, the brainstem anatomically includes three parts, from top to bottom namely the midbrain, the pons, and the medulla oblongata. Most previous studies emphasized the critical role of upper brainstem including midbrain and pons with its connection to cortical networks through serotonergic, dopaminergic, and noradrenergic neurotransmission in the pathophysiology of depression (Nye et al., 2013; Hahn et al., 2014; Smith, 2015; Numasawa et al., 2017; Wagner et al., 2017; Post and Warden, 2018). Unlike the midbrain and pons, the medulla is a more primitive location controlling low-level autonomic functions such as breathing, blood pressure and heart rate (Smythies, 2011) and was one part of the human central homeostatic network (CHN) (Edlow et al., 2016). Therefore, to identify aberrant interconnections between brainstem subregions and cortical networks at fine-grained level may contribute to a better understanding of the mechanism of onset of MDD.

In the present study, we investigated the functional connectivity pattern alterations of brainstem subregions using rs-fMRI in a group of 23 medication-free MDD patients and 34 gender-, age-, and education level-matched healthy controls (HC). We first defined three subregions of brainstem, namely the midbrain, pons and medulla separately using a recently developed brainstem atlas (Iglesias et al., 2015). Then, whole brain RSFC analyses were performed to identify the changed functional connectivity patterns for each brainstem subregion in MDD patients. According to previous findings on disrupted functional connectivity of brainstem and its subregions in depression (Smith, 2015; Wagner et al., 2017), we hypothesized that there might be also dysfunctions within the functional network based on the three subregions of brainstem in medication-free MDD patients.

## Materials and Methods

### Subjects

Twenty three medication-free, right-handed MDD patients and 34 age-, gender-, and educational level- matched healthy controls (18–46 years) were recruited from the Affiliated Brain Hospital of Guangzhou Medical University (Table 1). The diagnosis of MDD used the Structured Clinical Interview based on DSM-IV criteria (SCID) and the Chinese version of 24-item Hamilton Depression Rating Scale (HDRS). Hamilton Anxiety scale (HAMA) was also used to assess their anxiety level. Additionally, HCs were screened with no Axis I Disorders based on the SCID non-patient edition. All the HCs reported no history of psychiatric illness for all biological relatives within three generations. Both MDD and HC groups reported no lifetime history of head injury, seizures, serious medical or surgical illness, as well as substance abuse, and were free of MRI contraindications. This study was approved by the local Ethics Committee of the Affiliated Brain Hospital of Guangzhou Medical University with written informed consent from all subjects and was carried out in accordance with their recommendations. All subjects gave written informed consent in accordance with the Declaration of Helsinki.

**Table 1:** Demographics and clinical characteristics of the subjects used in the present study.

Subjects	MDD	HC	*p*-value
Number of subjects	23	34	
Gender (male: female)	9/14	15/19	0.7083
Age (mean ±*SD*)	30.48 ± 7.13	29.71 ± 7.09	0.6888
Years of education (mean ±*SD*)	13.35 ± 3.89	14.18 ± 2.17	0.3072
HDRS scores (mean ±*SD*)	34.30 ± 7.58		
HAMA scores (mean ±*SD*)	24.36 ± 8.63		
Age of onset (years)	27 ± 7.44		
Duration of illness (months)	43.04 ± 58.18		
Episodes (n, patients)			
First	17		
Recurrence	6		
Family history of MDD (n, patients)	5		

*Pearson’s chi-squared test was used for gender comparison. Two-sample t-tests were used for age and education comparisons. HDRS, hamilton depression rating scale; HAMA, hamilton anxiety scale.*

### Resting-State fMRI Data Acquisition

Resting-state fMRI data were acquired on a 3 Tesla MR imaging system (Philips Medical Systems, Best, Netherlands) with an eight-channel SENSE head coil in the Affiliated Brain Hospital of Guangzhou Medical University, Guangzhou, China, using a gradient-echo echo-planar imaging (GRE-EPI) sequence sensitive to blood oxygenation level-dependent (BOLD) contrast. Before scanning, tight and comfortable foam paddings and earplugs were used to reduce head moving and noise in the scanner separately. During the scanning, subjects were instructed to close their eyes but not to sleep. The acquisition parameters were as follows: repetition time (TR) = 2000 ms, echo time (TE) = 30 ms, flip angle (FA) = 90°, matrix = 64 × 64, field of view (FOV) = 220 × 220 mm^2^, 33 axial slices, slice thickness = 4 mm, inter-slice gap = 0.6 mm, 240 volumes.

### Resting-State fMRI Data Preprocessing

The resting-state fMRI data were preprocessed using Statistical Parametric Mapping (SPM8^1^) software and DPARSF (version 2.3 ^2^). It was started with discard of the first 10 volumes, slice timing, realignment based on the first volume for head motion correction, and followed by normalization based on MNI space template with 3 mm cubic voxel resolution, smoothing with a Gaussian kernel of 6 mm full-width at half maximum (FWHM), as well as regression of six motion parameters, white matter, and cerebrospinal fluid signals, and finally ended by filter with a temporal band-path of 0.01–0.1 Hz. After realignment, data with head-movement exceeded 1.5 mm of translation or 1.5 degrees of rotation in any direction was discarded. Moreover, “scrubbing” method was also used to eliminate the bad images based on the pre-set criteria (frame displacement, FD < 0.5), but no frame was deleted (FD < 0.3). Given the whole-brain signal regression exaggerates anti-correlation and to ensure the reliability of the obtained results, the global signal was not regressed (Wang et al., 2017a,b).

### Definition of Brainstem Subregions

**FIGURE 1:**
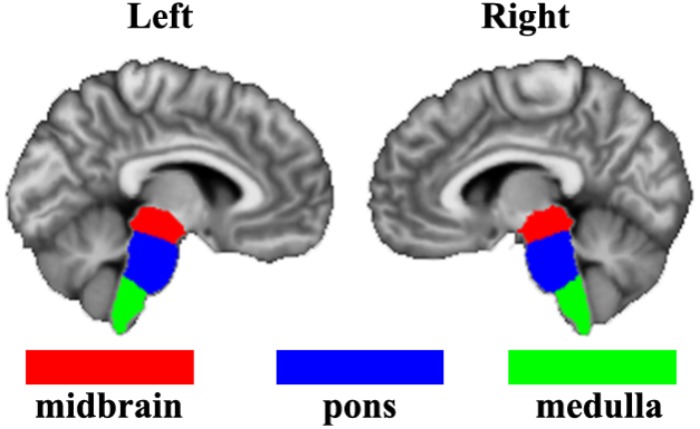
The definition of brainstem subregions based on a recent brainstem atlas which was constructed using Bayesian segmentation approach in MRI. Three subregions including midbrain, pons, and medulla were used in the present study.

The bilateral brainstem subregions were defined based on a recent brainstem atlas which was constructed using Bayesian segmentation approach in MRI (Iglesias et al., 2015). In this atlas, brainstem was symmetrically segmented into 4 subregions in each hemisphere, namely the midbrain, pons, medulla oblongata, and superior cerebellar peduncle. Given the superior cerebellar peduncle is too small and the smoothing effects of fMRI images, we did not include the superior cerebellar peduncle in our current study (Figure 1).

### Functional Connectivity Analyses

To identify the changed functional connectivity patterns of the brainstem subregions between MDD and HC, the whole brain functional connectivity analysis of each brainstem subregion was performed. We first extracted the mean time series of the brainstem subregions. Next, the functional connectivity was measured using Pearson’s correlations between the averaged time series of the brainstem subregions and voxels in the rest of the brain and Fisher’s z transformation was applied to normalize the functional connectivity maps. Two-sample *t*-tests (gender, age, and education as covariates) were performed to determine areas with significantly different functional connectivity to the brainstem subregions between MDD and the healthy controls. The significance was determined by a cluster-level Monte Carlo simulation (5000 times) using the updated Alphasim correction with corrected threshold of *p* < 0.05 (cluster-forming threshold at voxel-level *p* < 0.001), and minimum cluster size of 47 voxels.

### Functional Characterization With BrainMap Database

To determine the functional roles of the brain regions showing changed functional connectivity, BrainMap database ^3^ was used to characterize the behavior of these areas. The behavioral domains were determined by examining which types of tasks were significantly associated with these areas. Functional characterization of these areas was determined using forward inferences (Bzdok et al., 2013). The significance was established using a binomial test with *p* < 0.05 false discovery rate (FDR) corrected for multiple comparisons.

### Correlation Analyses

To determine the relationships between resting-state functional connection and HDRS, HAMA scores, correlation analyses were performed in MDD patients. The threshold of significance was set at *p* < 0.05.

## Results

### Demographics and Clinical Characteristics

The demographics and clinical characteristics of the subjects were shown in Table 1. No significant differences of gender (*p* = 0.81), age (*p* = 0.92), or education level (*p* = 0.17) were observed between MDD and HC groups.

### Disrupted Functional Connectivity of Brainstem Subregions

**FIGURE 2:**
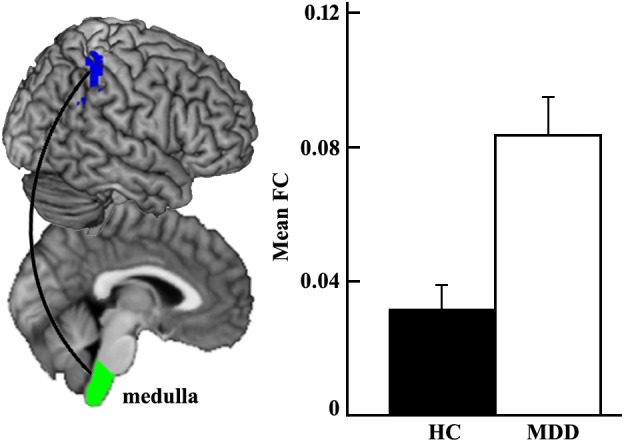
Increased functional connectivity between left medulla and right inferior parietal cortex (IPC) in MDD patients compared to healthy controls using two-sample *t*-test.

The whole brain functional connectivity analysis was performed for each brainstem subregion, and only increased functional connectivity between left medulla and right inferior parietal cortex (IPC) was found in the MDD group compared with the HC group (Figure 2).

### Functional Characterization Using Meta-Analysis

**FIGURE 3:**
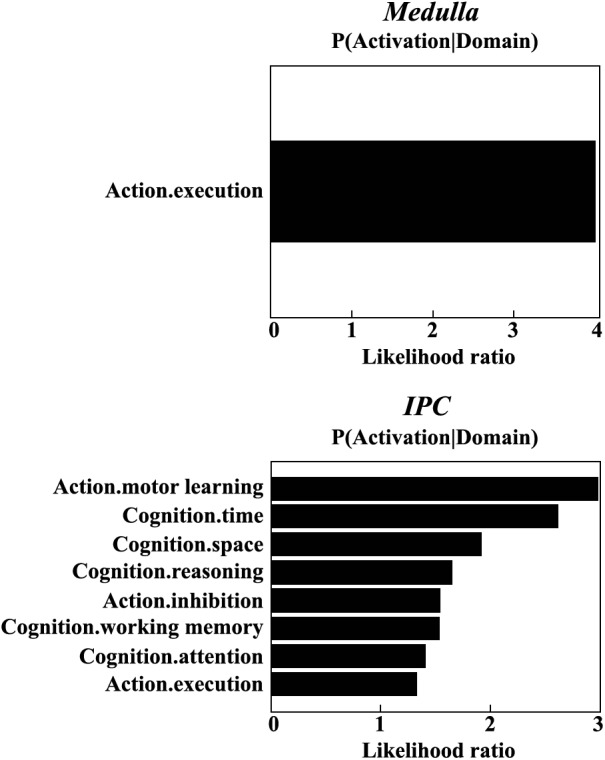
Functional characterization identified a significant association of the left medulla with action execution, and significant associations of right IPC with motor learning, execution, inhibition, and cognition of time, space, reasoning, working memory, as well as attention.

The meta-analysis on functional characterization of the left medulla was significantly involved in action execution, while that of right IPC was significantly associated with motor learning, execution, inhibition, and cognition of time, space, as well as reasoning. Moreover, functional characterization of IPC also identified its association with working memory and attention (Figure 3).

### Correlations With Clinical Characteristics

**FIGURE 4:**
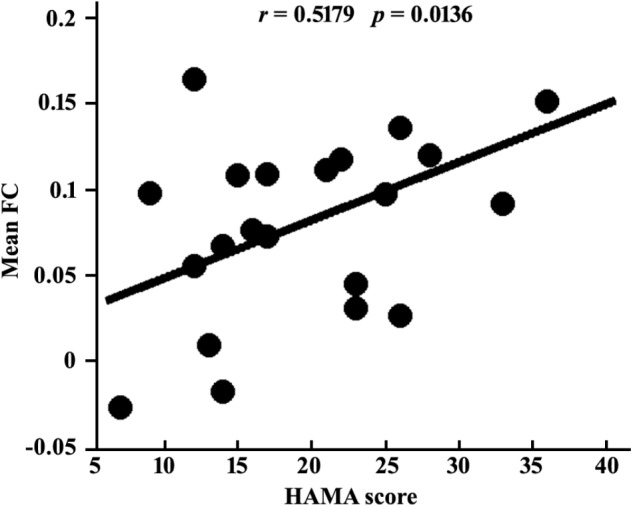
Positive correlation between mean resting-state functional connection of left medulla-right IPC and HAMA scores in MDD patients.

Given the normal distribution of both HDRS and HAMA scores in patient group shown by Shapiro-Wilks test (both *p*s > 0.2), Pearson correlations were used to determine the associations between the functional connectivity and scales. The result showed a significant association between the mean RSFC of left medulla – right IPC and HAMA scores (*r* = 0.518, *p* = 0.014) (Figure 4), but not with HDRS (*r* = 0.183, *p* = 0.403) in MDD patients.

## Discussion

In the present study, we investigated the functional connectivity patterns of brainstem subregions in medication-free MDD patients. Compared to healthy controls, we found increased functional connectivity between the left medulla and right inferior parietal cortex/lobule (IPC/IPL) in MDD. The changed functional connectivity showed significantly positive correlation with HAMA scores, suggesting higher functional connectivity between the left medulla and right IPC associates higher anxiety.

The increased functional connectivity between left medulla to cortical IPC/IPL in MDD patients was found. Previous studies found the associations between medulla and depression whereas they mostly focused on cardiovascular (Geraldes et al., 2016; Tsai et al., 2016) or central respiratory depression (Kashiwagi et al., 2011). However, increasing studies have indicated the important role of the interconnection from the medulla to limbic system including the amygdala, hippocampus, hypothalamus, insula, etc., involved in higher-level functions, such as mood control (Smythies, 2011), and conditioned fear expression (Vianna et al., 2008), response (Yoshida et al., 2014) as well as extinction (Rosa et al., 2014). A recent review demonstrated that almost all levels of the brainstem are involved in emotion, including regions in the medulla such as nucleus of the tractus solitaries in ascending emotional sensory network, rostral ventrolateral medullary nuclei and dorsal motor nucleus of the vagus nerve in descending emotional motor network, and caudal raphe nuclei in both descending and modulatory network (Venkatraman et al., 2017). Therefore, the medulla may also be an indispensable part of depressive disorder based network.

We found the role of the left medulla in depression through its connection to the right IPC/IPL. The IPL is a critical node in an integrative multiple networks such as DMN (Wang et al., 2012, 2016, 2017c; Guo et al., 2013; Mulders et al., 2015), CEN (Ellard et al., 2018), and the cognitive control network (CCN) (Crane et al., 2016; Stange et al., 2017), playing an important role in emotion, cognition and behavior. For example, previous studies showed a causal role of IPL with its transcranial magnetic stimulation (TMS) in enhanced processing of fearful body expressions (Engelen et al., 2015). IPL is also activated during self-face processing, and the intensity increased with subject age (Morita et al., 2018). Thus the dysfunction of IPL was obviously and frequently reported in MDD, such as increased fractional amplitude of low-frequency fluctuation (fALFF) values (Yamamura et al., 2016) and lower ReHo values (Lai and Wu, 2016) of the IPL, and decreased functional connectivity between the cerebellar and IPL, with the treatment-resistant depression (TRD) group decreased more than the sensitive (Guo et al., 2013). MDD also exhibited higher connectivity between the dorsal agranular insula and IPL compared with HCs (Wang C. et al., 2018). Even in the subclinical depression participants, positive correlations were observed between left IPL activity and happy attentional biases, suggesting an active coping attempt (Dedovic et al., 2016). Moreover, antidepressant drug treatment would lead to decreased activation in IPL, suggesting a restored deactivation of the DMN (Delaveau et al., 2011). The right IPL was indicated to be involved in interoception, execution, attention, action inhibition, social and spatial cognition, etc., and more implicated in visuospatial attention processing than the left one (Wang et al., 2016). Therefore, the impaired connection between the left medulla and right IPL may be associated with the neuropathology of MDD.

Indeed, this is confirmed by the functional characterization using meta-analysis, which showed medulla and IPC in motor learning, execution, inhibition, and cognition. The increased functional connectivity between both regions may indicate the dysfunctions of these fields in MDD patients, especially cognition such as working memory and attention. These deficits have been frequently reported in previous studies in MDD patients (Mulders et al., 2015; Wang J. et al., 2018). Our functional characterization findings provide further evidence for the important role of the connection between the medulla and IPC in the pathology of MDD.

We also found significant positive associations between HAMA scores and functional connectivity of left medulla and right IPC, indicating that down-regulation of their functional connectivity may decrease anxiety symptoms of MDD. Previous studies found increased ALFF in the right IPL (Yuan et al., 2018) and enhanced functional connectivity between periaqueductal gray and IPL (Arnold Anteraper et al., 2014) in social anxiety disorder (SAD) patients as compared to healthy controls. Also, the activation of IPL may differentiate the comorbid MDD and anxiety (MDD + Anx) patients to MDD patients to some extent in cognitive tasks such as Go/No-Go task (Crane et al., 2016). Research also demonstrated the important role of cognitive behavioural therapy (CBT) in SAD mainly via the brain activations of emotional response to and regulation of social criticism which both include IPL activation (Goldin et al., 2014). Even though the RSFC study found the biotypes for depression, it also overlapped with generalized anxiety disorder (GAD) at a very high proportion (Drysdale et al., 2017; Wager and Woo, 2017). Therefore, the present correlation finding may suggest anxiety-related symptoms in depression and provide a potential neural marker of distinguishing the depression and anxiety.

There are some limitations in the present study. First, only 23 MDD patients were used to investigate changes of the functional connectivity patterns of brainstem subregions. Therefore, the validity of the findings should be further tested in a larger sample. Second, as shown by Table 1, there is a wide range of the course of disease for MDD patients, which may have some impact on the results of functional connectivity. Third, the exact roles of the increased functional connectivity between medulla and IPC playing in depression needs to be further examined by task-related fMRI studies.

## Conclusion

The present study examined the abnormality of functional connectivity patterns of brainstem subregions and revealed increased functional connection between left medulla and right IPC in MDD patients compared to healthy controls. This finding indicates the functional abnormality in the early primary automatic functional system in MDD patients, which may facilitate the future early diagnosis for MDD.

## Author Contributions

JW and HW designed and supervised the study. HW and FW collected the data. LL, KW, YL, SG, XK, and FL analyzed the data. LL, KW, YL, HW, and JW drafted the manuscript. All authors discussed the results and commented on the manuscript.

## Conflict of Interest Statement

The authors declare that the research was conducted in the absence of any commercial or financial relationships that could be construed as a potential conflict of interest.

## Footnotes

1 http://www.fil.ion.ucl.ac.uk/spm

2 www.restfmri.net/forum/DPARSF

3 www.brainmap.org
